# A combination of methotrexate and zoledronic acid prevents bone erosions and systemic bone mass loss in collagen induced arthritis

**DOI:** 10.1186/ar2877

**Published:** 2009-12-10

**Authors:** Benoit Le Goff, Elise Soltner, Céline Charrier, Yves Maugars, Françoise Rédini, Dominique Heymann, Jean-Marie Berthelot

**Affiliations:** 1 INSERM UMR-S 957, 1 rue Gaston Veil, 44035, Nantes Cedex 1, France; 2 Université de Nantes, Nantes atlantique universités, Laboratoire de Physiopathologie de la Résorption Osseuse et Thérapie des Tumeurs Osseuses Primitives, EA3822, 1 rue Gaston Veil, 44035, Nantes Cedex 1, France; 3 Rheumatology Unit, Hôtel-Dieu, Nantes University Hospital, 1 place Alexis Ricordeau, 44093, Nantes Cedex 1, France; 4 Pôle de Biologie, Hôtel Dieu, Nantes University Hospital, 1 place Alexis Ricordeau, 44093, Nantes Cedex 1, France

## Abstract

**Introduction:**

Osteoclasts play a key role in the pathogenesis of bone erosion and systemic bone mass loss during rheumatoid arthritis (RA). In this study, we aimed to determine the effect of methotrexate (MTX) and zoledronic acid (ZA), used alone or in combination, on osteoclast-mediated bone erosions and systemic bone mass loss in a rat model of collagen induced arthritis (CIA). We hypothesized that MTX and ZA could have an additive effect to prevent both bone erosion and systemic bone loss.

**Methods:**

Arthritis was induced in 64 female Sprague-Dawley rats. After the clinical onset of CIA, rats were assigned to treatment with MTX (1 mg/kg/week), ZA (100 μg/kg twice weekly), both treatments at the same regimens, or vehicle. Arthritis score and paw thickness were recorded twice weekly. The rats were sacrificed on D28 and hind paws were removed for radiographic, histological and immunohistochemical analysis. The effects of treatments on osteoclastogenesis were determined by Tartrate resistant acid phosphatase (TRAP) staining. Micro-CT of the tibia was carried out for histomorphometric analysis. Bone mass density was evaluated by densitometry.

**Results:**

MTX significantly decreased the severity of CIA, whereas ZA slightly exacerbated it. When these two drugs were used in combination, MTX prevented the pro-inflammatory effect of ZA. The combination of ZA with MTX was more effective than MTX alone for reducing structural joint damage with a dramatic decrease of osteoclasts' number in the eroded joints. However, MTX alone also significantly reduced the number of osteoclasts and the number of CD68+ mononuclear cells. ZA alone, or ZA with MTX, significantly increased the systemic bone mass density measured by densitometry and bone volume on histomorphometric analysis.

**Conclusions:**

A combination of MTX and ZA prevented both bone erosion and systemic bone loss in a rat model of arthritis. Both treatments independently decreased the number of osteoclasts in the eroded joint. However, while MTX probably acts mainly through a decrease of inflammation, ZA has a direct effect on osteoclasts, allowing a dramatic down-regulation of these cells in inflamed joints. These two different mechanisms of action provide support for the use of a combination of these two drugs to improve the prevention of structural joint damage in RA.

## Introduction

Rheumatoid arthritis (RA) is characterized by a chronic inflammation of synovium, leading to progressive joint destruction. Erosions of the periarticular bone, the most specific hallmark of the disease, produce deformation, laxity, and functional disability. Local and systemic inflammation also favors generalized osteopenia or osteoporosis. Osteoclasts are considered as the principal cell type responsible for focal bone resorption in RA [1,2]. Gravallese and colleagues first described tartrate resistant acid phosphatase (TRAP) positive multinucleated cells in resorption lacunae at the bone-pannus interface in patients with juvenile arthritis [3]. Several lines of evidence have since confirmed the role of osteoclasts in bone destruction during RA. Osteopetrotic mouse models with a genetic block in osteoclast formation, such as receptor activator of nuclear factor kappa B-ligand (RANK-L) -/- mice, develop arthritis but display no bone erosion [4]. Treatment with a chimeric osteoprotegerin fusion protein, which inhibits osteoclast differentiation, efficiently prevents bone erosion in the rat collagen-induced arthritis model [5]. The origin of osteoclasts in arthritic joints remains unclear. These cells may differentiate from monocytic precursor cells that home to the inflamed synovial tissue or from bone marrow precursors, under the influence of cytokines, such as RANK-L or TNF-alpha, generated in the synovium of patients with RA [6]. Transdifferentiation from other subsets of immune cells, including dendritic cells, has also been proposed [7].

Osteoporosis in RA patients may be attributed to various risk factors, including primary osteoporosis risk factors, immobilization, use of corticosteroids, and systemic inflammation. Osteoclasts also play a crucial role in the development of generalized osteoporosis, mediated through the osteoprotegerin/RANK/RANK-L signaling system [8]. Recent studies have demonstrated that targeting RANK-mediated osteoclastogenesis with denosumab prevents systemic bone loss in RA patients [9].

The prevention of joint damage and systemic bone mass loss is a key goal of treatment for RA. Zoledronic acid (ZA), a nitrogen-containing third-generation bisphosphonate, is widely used to treat metastatic bone disease and has recently been used for osteoporosis [10,11]. ZA, like other bisphosphonates, has a direct effect on mature osteoclasts, inducing their apoptosis and inhibiting their activity. ZA has been shown to be effective for the prevention of osteoporosis, but its ability to confer local joint protection remains a matter of debate. Indeed, although ZA has been shown to prevent bone erosion in animal models of arthritis [12,13], only one study in humans has reported a significant decrease in bone erosion in patients in the early stages of RA treated with ZA [14].

Methotrexate (MTX) is the first-line therapy for RA. It is effective against inflammatory symptoms but also in the prevention or reduction of bone erosions [15]. It is also required to achieve maximal suppression of bone destruction during the treatment of RA patients with TNF-alpha-inhibitor [16]. Although TNF inhibitors have clearly improved the prevention of joint destruction, double-blind comparisons of MTX and TNF inhibitors, used alone and in combination, have shown the structural benefits of MTX treatment to be very similar to the effect of TNF inhibitors alone [17]. The underlying mechanism of the strong structure-modifying effect of MTX has not been studied in detail. MTX may inhibit osteoclastogenesis indirectly, by decreasing the production of osteoclastogenic cytokines, such as TNF-α and IL-6, or by reducing RANK-L secretion by synovial fibroblasts or macrophages [18]. Alternatively, low-dose MTX may inhibit osteoclastogenesis directly, although only high doses of MTX have been reported to have negative effects on bone, probably through the inhibition of osteoblasts [19].

Very few studies have investigated the effect of MTX *in vivo*, even for osteoclastogenesis. We therefore sought to determine the effects of MTX and ZA, used alone or in combination, on bone erosions and systemic bone loss in the rat collagen-induced arthritis model, focusing on the effect of these treatments on osteoclasts. In addition to clinical and radiological evaluations, we also investigated the effect of MTX and ZA on the number of osteoclasts in the eroded joint. We also studied CD68+ cells, because monocytes may be modulated by MTX therapy and a subset of these cells a source of osteoclast precursors. Systemic bone mass was analyzed by densitometry and histomorphometry was investigated by micro-computed tomography (CT). We hypothesized that MTX and ZA would have additive effects, decreasing bone erosion and bone mass loss.

## Materials and methods

### Animals and induction of collagen-induced arthritis

We used eight-week-old female Sprague-Dawley rats (IFFA-CREDO, l'Arbresles, France) as a model of arthritis. All animals were fed standard rodent chow and supplied with drinking water *ad libitum*. Animals were allowed to acclimatize to the conditions for one week before the experiments. Arthritis was induced in all the rats as follows: lyophilized native bovine type II collagen (Sigma, Lyon, France) was dissolved at a concentration of 2 mg/ml in 0.1 M acetic acid. The solution was incubated overnight at 4°C, and 0.3 ml of this solution in a 1:1 emulsion with complete Freund's adjuvant was injected intradermally into the base of the tail on days 0 and 14. Arthritis developed 15 to 17 days after the first injection. All experimental procedures conformed to institutional guidelines, were approved by the institutional review board and were carried out under the supervision of accredited investigators (FR, DH).

### Treatments

Two independent experiments were performed. In each experiment, 32 rats were assigned to four groups of eight rats each and treated with MTX (Sigma, Lyon, France), ZA (Novartis Pharma, Bâle, Switzerland), MTX + ZA or placebo. Group 1 was treated with MTX dissolved in PBS at a dose of 1 mg/kg/week, administered intraperitoneally in a single installment. Group 2 received 100 μg/kg ZA, administered subcutaneously, twice weekly. Group 3 received both treatments at the same doses as used for groups 1 and 2. Group 4 was used as the positive control and treated with PBS. All the treatments were used at doses previously described in the literature as being effective [12,13,20]. Treatments were started at the onset of arthritis, 15 days after the first injection.

### Clinical assessment of arthritis

Clinical signs of arthritis were assessed by investigators blind to the treatment group, twice weekly, as previously described [1]. Joint swelling was assessed with a semi-quantitative clinical score running from 0 to 4 (0 = no swelling, 1 = weak swelling and/or erythema, 2 = mild swelling, 3 = moderate swelling, 4 = severe swelling of the toes and ankle). This system yielded a total weekly score between 0 and 8 for each animal (sum of two assessments per week, each of which gave a score of 0 to 4). Hind footpad width was also measured with calipers at baseline, twice weekly. The body weight of the rats was monitored with a balance with a precision of 0.1 g.

### Radiological examinations

At the end of the experiment, rats were sacrificed using carbon dioxide asphyxiation and the hind paws were radiographed with a digital mammography system (Planmed, Helsinki, Finland) used to provide high-resolution images. The ankle and tarsus joints were graded for erosions (0 to 3) and soft-tissue swelling (0 to 3), with scores of 1 and 3 corresponding to no involvement and to extensive involvement, respectively [21]. Two observers blind to treatment assignment and with significant experience in reading and rating radiographs for patients with RA evaluated the radiographs. A total radiological score was obtained by summing the scores awarded to the two hind paws by both observers, giving a maximum score of 12 per rat for each radiological parameter.

### Conventional histology, immunohistochemistry and TRAP staining

After radiological examination, the hind paws were removed, fixed by incubation in 10% buffered formaldehyde for 48 hours and decalcified by incubation in 4.1% EDTA, 0.2% Paraformaldehyde (PFA) in PBS at 4°C, with the decalcifying solution changed twice weekly, for four weeks. The paws were embedded in paraffin, serial sections (5 μm) were cut and mounted on glass slides and stained with H&E. Histological analysis (three sections per rat) were carried out for the tibiotarsal and all intertarsal joints. For standardization, tibial, tarsal and metatarsal bones had to be present in the same section for analysis. Inflammation was quantified on H&E-stained sections, using a semi-quantitative score (scale of 0 to 3) at low magnification (×10): 0 = normal, 1 = mild inflammation, 2 = moderate inflammation, 3 = marked inflammation [22]. Bone erosion was scored at low (× 10) and high (× 40) magnification, on the following scale: 0 = none, 1 = mild, 2 = moderate and 3 = severe erosions. For TRAP staining, sections were incubated for one hour in 1 mg/ml naphthol AS-TR phosphate (N-(4-Chloro-2-methylphenyl)-3-(phosphonooxy)naphthalene-2-carboxamide), 60 nmol/l NN-dimethylformamide, 100 nmol/l sodium tartrate, and 1 mg/ml Fast red TR salt solution (all from Sigma, Lyon, France) The number of osteoclasts (three or more TRAP-positive cells) within the eroded area was determined at high magnification (× 100), with a commercial image analysis program (Axiovision 4.7, Zeiss, Le Pecq, France). The results are expressed as the total number of osteoclasts per slide.

We investigated the effect of the treatments on monocyte infiltration, by carrying out immunohistochemical analyses with an automatic tissue stainer (Autostainer 360, Lab vision, Fremont, CA, USA), using a monoclonal mouse anti-rat CD68 antibody (Clone ED-1, 1:100 dilution, abd Serotec, Cergy Saint-Christophe, France). Endogenous peroxidase activity was blocked by incubation for 10 minutes in 3% hydrogen peroxide in PBS. Sections were blocked by incubation with 4% BSA in PBS and were then incubated for one hour at room temperature with the primary antibodies. Biotin-conjugated anti-mouse secondary antibodies (Vector, Burlingame, CA, USA) were used at a dilution of 1:250. They were incubated with the sections for one hour at room temperature. The sections were then incubated with streptavidin peroxidase for 30 minutes and antibody binding was detected with the AEC staining kit (Sigma, Lyon, France), the antigen-expressing cells being stained brown. The number of CD68-positive cells per unit area of inflammation was determined with commercial image analysis software (Axiovision 4.7, Zeiss, Le Pecq, France). Results are expressed as the number of cells per cm^2 ^of inflammation.

### Dual-energy X-ray absortiometry and micro-CT analysis

Dual-energy X-ray absortiometry (DXA) was performed at the end of the experiment, on intact animals, using a LUNAR Prodigy Advance densitometer (General Electric, Madison, WI, USA) calibrated for small animals. Each rat was placed in a prone position. Several measurements were taken: total body weight (with % of fat mass); bone mineral content and bone mineral density (BMD) of the femurs and lumbar spine of each treated rat. Architectural parameters were analyzed by high-resolution X-ray micro-CT, using the SkyScan-1072 (SkyScan, Aartselaar, Belgium) system for small-animal imaging. Each tibia was scanned parallel to its longitudinal axis (60 kV, 148 μA). A core of 200 slides, each 11 μm thick (7 mm long) was used for bone morphometry evaluations with SkyScan CtAn software. The following factors were measured: total volume, bone volume (BV) and the BV/tissue volume (TV) ratio. Trabecular BV and cortical BV were evaluated separately and the ratio of these two volumes was calculated. Trabecular bone thickness, trabecular number and separation were measured with a semi-automatic morphing procedure, from total BV. Cortical thickness was evaluated in the middle of the image and was assessed three times, on five separate slides.

### Statistical analysis

All results are expressed as means +/- standard error of the mean. Mann-Whitney U tests were used to compare group mean values, in GraphPad Prism version 5 software (GraphPaD Software, San Diego, CA, USA). *P *values less than 0.05 were considered significant.

## Results

### Effect of MTX and ZA on the collagen-induced arthritis disease course

**Figure 1:**
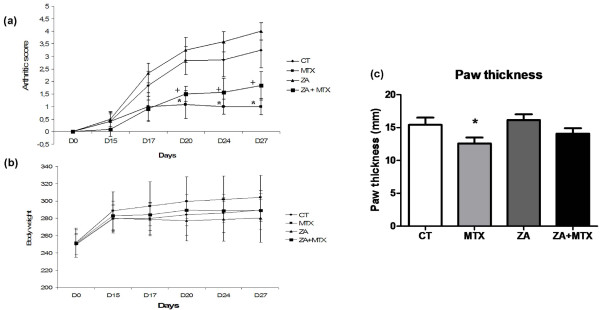
Clinical course of collagen-induced arthritis. **(a) **Arthritis score was evaluated at day 0 and day 14 (first and second collagen injections) and then every three days after disease onset (days 17, 20, 24 and 27). Methotrexate (MTX) treatment resulted in a significantly lower arthritis score than recorded for the control or zoledronic acid (ZA) groups. Arthritis score was also decreased by ZA + MTX, but the difference with the control group was not statistically significant. ZA had a slight pro-inflammatory effect, whether used alone or in combination with MTX. **(b) **Body weight (g) measured at each clinical evaluation. After arthritis induction, the body weight reached a plateau from day 15 with no significant difference between all groups assessed. **(c) **Paw swelling in rats was assessed by caliper measurement at termination according to the treatments. MTX resulted in a significantly greater reduction in paw thickness at the end of the experiment. No such effect was observed with ZA or ZA + MTX. *P < 0.05 versus CT group; +*P *< 0.05 versus ZA group.

We first addressed the effects of MTX and ZA, used alone or in combination, on the clinical course of arthritis in rats. From day 20 to the end of the experiment, MTX treatment resulted in an arthritis score significantly lower than those for the control and ZA groups (Figure 1a). When used alone, ZA had a slight, but non-significant, pro-inflammatory effect, resulting in a higher arthritis score than for the control group. The combination of MTX and ZA also attenuated the increase in total arthritis score, but to a lesser extent than MTX alone. At the end of the experiment, paw thickness was significantly lower only in the MTX group (12.6 mm versus 15.5 mm in the control group (*P *< 0.05); Figure 1c). The most severe exacerbations were detected in rats treated with ZA (paw thickness of 16.2 mm). Rats treated with ZA + MTX had slightly thinner paws (14 mm), but the difference in paw thickness between these rats and those of the control group was not statistically significant. Body weight was measured at each clinical evaluation. After arthritis induction, the body weight reached a plateau from day 15 in all groups assessed (Figure 1b).

### Radiological examination

**Figure 2:**
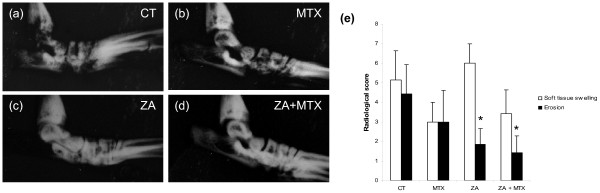
Representative plain radiographs of the hind paws obtained at the end of the experiment. The **(a) **control, **(b) **methotrexate (MTX), **(c) **zoledronic acid (ZA), and **(d) **ZA + MTX groups are shown. Severe loss of subchondral bone was seen in the control group, with multiple areas of erosion, whereas lower levels of destruction were observed in MTX-treated rats. ZA completely prevented bone destruction. **(e) **Soft-tissue swelling and erosion radiographic score for the ankle and tarsus joints. ZA alone or in combination with MTX significantly decreased the erosion score, but had a pro-inflammatory effect, increasing soft-tissue swelling. MTX also decreased the number of areas of erosion and decreased the soft-tissue swelling. *P < 0.05 versus computed tomography (CT) group.

The effects of treatments on local inflammation and bone erosion were assessed by plain X rays of the hind paws at the end of the experiment, with the measurement of soft-tissue swelling and the degree of bone erosion. ZA treatment, alone or in combination with MTX, significantly decreased erosion score (1.85 ± -0.8 (*P *< 0.05), 1.45 ± -0.9 (*P *< 0.05), respectively, versus 4.4 ± -1.5 for the control group; Figure 2e). MTX also decreased the number of erosions, but to a lesser extent than ZA (3 ± -1, *P *= not significant versus control). Conversely, MTX decreased the soft-tissue swelling score, whereas ZA did not (3 ± -0.7 versus 5.1 ± -0.7). Treatment with a combination of MTX and ZA decreased inflammation, but inflammation levels remained slightly higher than with MTX alone (3.4 ± -0.6). Inflammation and erosion were clearly dissociated in the ZA-treated group, whereas these two effects were coupled in the MTX and control groups.

### Effects of treatments on inflammation and CD68+ cells

**Figure 3:**
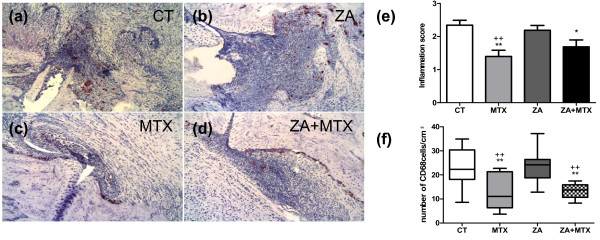
Representative histological profiles of rat ankle joints at the end of the experiment. The **(a) **control, **(b) **methotrexate (MTX), **(c) **zoledronic acid (ZA), and **(d) **ZA + MTX groups are shown (×10). Immunohistochemical staining, with CD68+ cells stained brown. **(e) **Histological inflammation score. MTX, whether used alone or in combination with ZA, significantly decreased the inflammation score, as shown by comparisons with the control group. **(f) **Number of CD68+ cells per unit area of inflammation. MTX, whether used alone or in combination with ZA, resulted in the presence of significantly fewer CD68+ cells than observed in the control and ZA groups. *P < 0.05 versus computed tomography (CT) group; **P < 0.005 versus CT group; ++*P *< 0.005 versus ZA group.

To address the effect of ZA and MTX on synovial inflammation we next performed a histological analysis of the joints (Figure 3). Histological inflammation scores were significantly lower in MTX-treated rats than in the control and ZA-treated groups (1.4 ± -0.2 versus 2.3 ± -0.14 (*P *= 0.005) and 2.2 ± -0.15 (*P *= 0.01), respectively). We found no difference in paw inflammation between the ZA and control groups. Treatment with a combination of MTX and ZA significantly decreased the inflammation score, but was less effective than MTX alone (1.6 ± -0.2, *P *= 0.04 versus control).

We then investigated the effect of treatment on CD68+ cells, which arise from the monocytic lineage and include potential osteoclast precursors. We considered only cells present in the inflammatory infiltrate, ignoring those on the surface of the bone, to avoid counting mature osteoclasts. MTX treatment, alone or in combination with ZA, resulted in the presence of significantly fewer CD68+ cells/cm^2 ^in the inflammatory infiltrate (Figure 3f) than were observed in the control group (12.78 ± -3 (*P *= 0.03) and 13.3 ± 1.4 (*P *= 0.02), respectively, versus 23.1 ± -2.5) or the ZA group (23.8 ± -2.5; *P *= 0.04 and *P *= 0.01, respectively). ZA had no effect on the number of CD68+ cells.

### Effects of treatments on histological erosion score and osteoclast number

**Figure 4:**
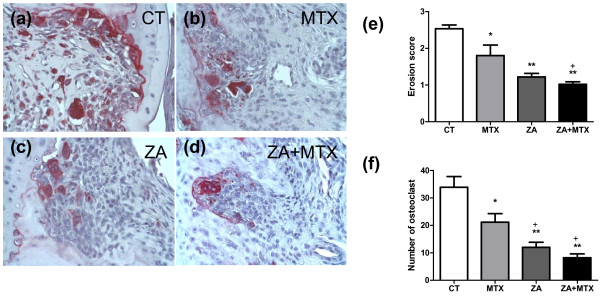
Representative TRAP staining profiles. The **(a) **control, **(b) **zoledronic acid (ZA), **(c) **methotrexate (MTX), and **(d) **ZA + MTX groups are shown (×40). **(e) **Erosion score. Bone erosion was strongly inhibited in the ZA group. MTX also significantly reduced bone erosion, although to a lesser extent than ZA. **(f) **Number of osteoclasts (multinucleated tartrate resistant acid phosphatase (TRAP) positive cells) at the bone/synovium interface in the eroded joint of the tibiotarsal and intertarsal joints. ZA, used alone or in combination with MTX, strongly decreased the number of TRAP-positive cells. MTX also decreased the number of TRAP+ cells in the eroded joint. *P < 0.05 versus computed tomography (CT) group; **P < 0.005 versus CT group; +*P *< 0.05 versus MTX group.

Histological analysis was carried out to confirm the effects of treatments on bone erosion observed on plain X-rays. Bone erosion, as assessed by a semi-quantitative score, was strongly inhibited in rats treated with ZA (1.2 ± -0.09 versus 2.5 ± -0.1 for the control group (*P *< 0.0001); Figure 4e). MTX also reduced significantly the erosion score compared with control, although this score remained higher than that for ZA (1.8 ± -0.3; *P *= 0.03). The combination of ZA and MTX was significantly more effective in the prevention of erosion than MTX alone (1.8 ± -0.3 versus 1.01 ± -0.07; *P *= 0.01), whereas there was no difference between the ZA + MTX and ZA groups (*P *= 0.1).

To assess the effect of ZA and MTX on the number of osteoclasts, joint sections were stained with TRAP. Only TRAP-positive multinucleated cells located at the bone surface within the bone erosions were considered to be osteoclasts. ZA, whether used alone or in combination with MTX, strongly decreased the number of osteoclasts in the areas of erosion. ZA treatment (alone or together with MTX) resulted in significantly fewer osteoclasts in these areas than observed in the control group and the group of animals treated with MTX alone (12 ± -1.8 and 8.2 ± -1.4 versus 33.9 ± -4 and 21.1 ± -3 respectively; Figure 4f). However, a significant decrease of osteoclast number was also present in MTX-treated group compared with the CT group (*P *= 0.04).

### Effects of treatments on bone density and micro-CT parameters

**Figure 5:**
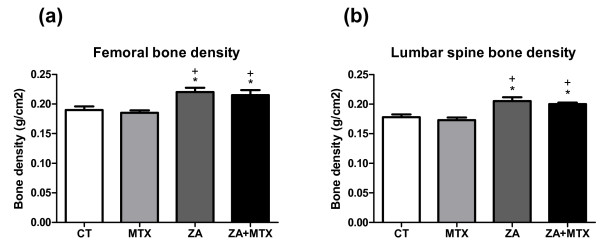
Bone density measured with a densitometer suitable for use with small animals. Zoledronic acid (ZA) significantly increased the bone density of the femur and spine in rats. We found no difference between the control and methotrexate (MTX) groups. Treatment with a combination of MTX + ZA was as effective as treatment with ZA alone. Values are the mean ± standard deviation. *P < 0.05 versus computed tomography (CT) group; +*P *< 0.05 versus MTX group.

We assessed the effects of treatments on BMD, by carrying out DXA on the right femurs and lumbar spine of rats at the end of the experiment. The BMD of the femur was significantly higher in rats treated with ZA or with ZA + MX than in the rats of the control group (+16%, *P *= 0.01 and +18%, *P *= 0.005, respectively; Figure 5). BMD was similar in the MTX and control groups (0.19 ± -0.006 versus 0.18 ± -0.01 g/cm^2^; *P *= 0.6). No difference in total body weight or percentage fat mass was found between the four groups (data not shown).

**Figure 6:**
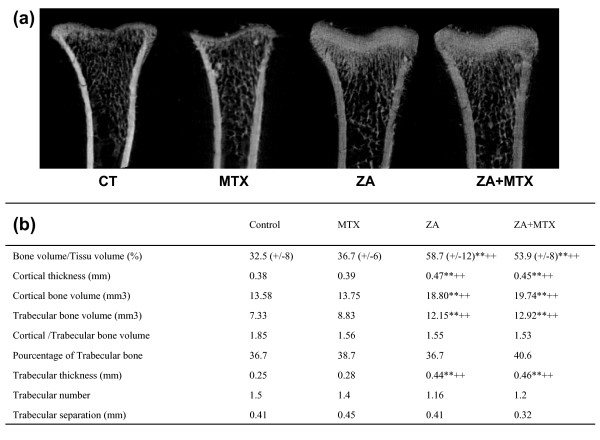
Micro-CT analysis. **(a) **Representative micro-computed tomography (CT) image of the distal tibia for each rat group. **(b) **Histomorphometric analysis of the distal tibia for the various treatment groups, showing a significant increase in bone volume (BV)/tissue volume (TV) ratio, cortical thickness, trabecular and cortical bone volume and trabecular thickness in rats treated with zoledronic acid (ZA). Methotrexate (MTX) had no effect on histomorphometric parameters. Values are the mean ± standard deviation. **P < 0.005 versus CT group; ++*P *< 0.005 versus MTX group.

The effects of the treatments on systemic bone loss were assessed by micro-CT evaluation of the proximal end of the left tibia with a quantitative histomorphometric imaging method (Figure 6). ZA treatment alone or in association with MTX significantly increased the ratio of BV/TV (58.7 ± -12 and 53.9 ± -3%) over that in the control (32.5 ± -4%; *P *= 0.03 and *P *= 0.01, respectively), whereas treatment with MTX alone did not (36.7 ± -3, *P *= 0.4). Cortical thickness was also increased by ZA treatment (0.47 mm versus 0.38 mm in the control group, *P *< 0.05), but not by MTX (0.39 mm). Trabecular and cortical bone volumes were increased by ZA, with no change in the cortical/trabecular volume ratio. Trabecular thickness was significantly greater in the groups treated with ZA or ZA + MTX than in the control group, whereas there was no significant difference between the group treated with MTX and the control group.

## Discussion

Bone erosions and generalized bone loss are among the most serious features of RA, leading to joint deformation, fractures, and severe disability. New evidence has confirmed that osteoclasts are key mediators of these two sorts of bone loss in RA. Traditional disease-modifying antirheumatic drugs (DMARDs) and biological treatments are capable of controling joint inflammation and bone erosions in most RA patients. However, some patients experience worsening structural joint damage despite apparently good control of disease activity. In such patients, a combination of MTX (and/or biological agents) with anti-resorptive drugs directly targeting pathogenic osteoclasts, might be a practical solution. We investigated this possibility, by comparing the clinical and structural efficacy of MTX and ZA, alone or as a combination, in a rat CIA model.

Our study findings confirm that MTX and ZA are efficient in the prevention of arthritic bone destruction. Indeed, both treatments significantly decreased radiographic and histological erosion scores in colagen-induced arthritis, with ZA the more effective of the two. In our experiments, ZA decreased the number of osteoclasts in the ankle, where a clear dissociation between inflammation and destruction was observed. ZA strongly and selectively down-regulated the number of TRAP+ cells but had no effect on the inflammatory infiltrate. This dissociation between bone protection and inflammation has been described before and indicates a direct effect of ZA on osteoclast numbers [12,13]. Osteoprotegerin, an inhibitor of osteoclastogenesis, also decreases the number of osteoclasts and gave similar results [5].

Despite experimental demonstrations of the efficacy of ZA for preventing bone erosion, the use of bisphosphonates in daily practice for the treatment of RA patients remains controversial. A single-center, proof-of-concept study of 39 patients receiving MTX by Jarrett and colleagues showed that addition of ZA to the treatment regimen decreased magnetic resonance imaging erosion scores by 61% at six months compared with MTX alone [14]. However, less potent bisphosphonates, such as clonodronate or pamidronate, prevent bone erosion only if used at high doses [23-25]. ZA is a third-generation bisphosphonate with an inhibitory effect on bone resorptive activity that are 100-fold to 10,000-fold stronger than those of the second and first generation. Moreover, a dose of 20 μg/kg is sufficient to prevent bone loss in ovariectomized rats [26]. The high dose of ZA used in our study and its powerful inhibitory effect on osteoclast-mediated bone resorption may account for the dramatic decrease in bone erosion observed. These results must be tempered by the consideration that ZA may have a proinflammatory effect and decrease the effectiveness of MTX. Indeed, we observed that ZA used alone or in combination with MTX had a slight but not significant pro-inflammatory effect with an increased arthritis score and paws thickness at the end of the experiment. However, bisphosphonates, such as alendronate or clonodronate, used in animal models of arthritis, demonstrated an anti-inflammatory effect [27]. These discrepancies are probably related to the difference of dose and type of bisphosphonate used. The potential proinflammatory effect and the risk of jaw osteonecrosis associated with the use of a high dose of bisphosphonate indicate that low dose of ZA would probably be the best option for RA patients.

The mechanisms responsible for structural joint protection by MTX are unclear and probably multifactorial. MTX has a powerful anti-inflammatory effect *in vivo *and inhibits human synovial fibroblast RANK-L production and osteoclastogenesis in a dose-dependent manner [18]. *In vitro*, MTX also abolishes the IL-6 synthesis stimulated by pro-inflammatory cytokines such as IL-17 and TNF-α in osteoblasts [28]. Furthermore, MTX promotes adenosine release *in vivo *and *in vitro *and adenosine strongly inhibits the monocyte fraction, which contains osteoclast precursors [29,30]. Thus, multiple mechanisms may explain the inhibitory effects of MTX on inflammatory bone destruction. In adjuvant arthritis, MTX increases the number of osteoclasts and pit formation in bone marrow cultures from non-arthritic rats, but has no effect on bone mass [31]. In arthritic rats, MTX attenuates arthritis and restored the decreased osteogenic activity of bone marrow cells, and reduced their increased bone resorptive activity to normal levels [31]. Similarly, and consistent with the inhibitory effects of MTX on monocytes, our study provides evidence that MTX reduces the number of CD68+ cells in the inflammatory infiltrate. Interestingly it also decreases TRAP+ (osteoclast) cell numbers in rats with collagen-induced arthritis. However, the precise effect of MTX on osteoclast precursors population needs to be clarified.

The skeletal effects of MTX are well known. High dosage of MTX has negative effects on bone mass in patients treated for malignancies [32], and the prolonged administration of low doses of MTX in rats causes significant osteopenia due to the inhibition of osteoblast activity and the stimulation of osteoclast recruitment, resulting in a net increase in bone resorption [33,34]. The bone disease induced by high doses of MTX is characterized by stress fractures, diffuse bone pain and osteoporosis [35]. However, possible bone toxicity of long-term, low-dose MTX treatment in humans remains a matter of debate and is difficult to demonstrate in cross-sectional [36,37] or longitudinal studies [38]. In our study, we found no difference between rats with collagen-induced arthritis given short-term MTX treatment and control rats in terms of BMD and histomorphometric analysis. Indeed, in our experiments, ZA increased BMD and improved all histomorphometric parameters. These results are consistent with those of Spadaro and colleagues, who found that alendronate prevented bone loss induced by high-doses of MTX treatment in rats [39]. Other bisphosphonates, such as alendronate, have also been shown to be effective in the prevention of systemic bone loss associated with RA during treatment with MTX [40] or corticosteroids [11]. Our experimental results suggest that treatment with a combination of ZA and MTX would have beneficial effects in many RA patients, by decreasing joint erosion and increasing bone mineral density.

## Conclusions

In summary, our study demonstrates that a combination of MTX and ZA prevented both bone erosion and systemic bone mass loss in the collagen-induced arthritis model of RA. In our study, ZA was more effective than MTX for preventing structural damage. MTX and ZA both decreased the number of osteoclasts in the eroded joint, but seemed to prevent bone erosion in different ways. ZA is known to inhibit osteoclast formation, function and survival. It therefore probably acts on mature osteoclasts, without reducing the number of osteoclast precursors. Our data suggest that MTX may also inhibit osteoclastogenesis. We observed that it decreases the number of CD68+ cells in the synovium. Further studies will be required to elucidate the precise effects of MTX on RANK+ precursor cells within the CD68+ monocyte fraction. These different mechanisms of action provide a rationale for combining these two commonly used drugs to improve the prevention of structural joint damage in RA. Randomized clinical trials comparing MTX used alone and MTX used in combination with ZA should be carried out to establish a rational treatment strategy for RA.

## Abbreviations

BMD: bone mineral density; BSA: bovine serum albumin; BV: bone volume; CT: computed tomography; DMARD: disease-modifying antirheumatic drugs; DXA: dual-energy X-ray absortiometry; H&E: hematoxylin and eosin; IL: interleukin; MTX: methotrexate; PBS: phosphate-buffered saline; RA: rheumatoid arthritis; RANK-L: receptor activator of nuclear factor kappa B-Ligand; TNF-α: tumor necrosis factor alpha; TRAP: tartrate resistant acid phosphatase; TV: tissue volume; ZA: zoledronic acid.

## Competing interests

JMB received fees from Nordic-pharma. All other authors declare that they have no competing interests.

## Authors' contributions

BLG conceived of the study idea, contributed to experimental design, performed experiments, and participated in the writing of the manuscript and data interpretation. ES and CC contributed to experimental design and assisted with animal studies and histological procedures. FR contributed to animals experimental design and supplied the Spargue-Dawley rats. JMB, YM and DH assisted with conception of the study idea and participated in its design, data analysis, and the writing of the manuscript.
